# Predictors of Custodial Grandparents’ Perceived Barriers to the Use of Services

**DOI:** 10.1093/geroni/igab046.3038

**Published:** 2021-12-17

**Authors:** Julian Montoro-Rodriguez, Bert Hayslip Jr, Jennifer Ramsey

**Affiliations:** 1 UNC Charlotte, Charlotte, North Carolina, United States; 2 University of North Texas, Denton, Texas, United States; 3 Case Western Reserve University, Cleveland, Ohio, United States

## Abstract

Getting timely access to help, information, and a variety of services is paramount among the challenges of raising a grandchild, and grandparents face a variety of internal and external barriers in getting such help. The present pilot exploratory study focused on caregiving-related and personal resource variables best predicting grandparent caregivers’ perceptions of barriers to receiving services. Fifty-two grandparents (M age = 59.1) raising their grandchildren completed measures assessing caregiver strain, social support, resilience, self-care, psychosocial adequacy, health, depression, and grandchild relationship quality. They also completed measures of the extent to which they faced personal and caregiving-related difficulties giving rise to the need for services (e.g. health, grandchild well-being, support from others) as well as the extent to which they had experienced barriers to service (health/financial limitations, isolation, transportation, respite care, lack of knowledge of services) in the past 3 months. Correlations (p < .05) suggested that psychosocial adequacy (r = -.32), depression (r = .27), caregiver strain (r = .42) and difficulties (r = .48) were all related to greater perceived barriers. Regression analyses (F7, 40 = 2.81, p < .02) indicated that caregiver strain (Beta = .33, p < .05) and difficulties giving rise to the need for services (Beta = .32, p < .04) emerged as most salient in predicting barriers. These findings underscore the fact that personal, caregiving-related, and interpersonal factors exacerbate the barriers associated with grandparents’ accessing needed services and reinforce such factors’ impact on grandparents as targets for overcoming impediments to accessing services among them.

